# Postnatal Depression Is a Public Health Nursing Issue: Perspectives from Norway and Ireland

**DOI:** 10.1155/2013/813409

**Published:** 2013-09-05

**Authors:** Kari Glavin, Patricia Leahy-Warren

**Affiliations:** ^1^Department of Nursing, Diakonova University College, Fredensborgveien 24 Q, 0177 Oslo, Norway; ^2^School of Nursing & Midwifery, Brookfield Health Sciences Complex, University College Cork, Ireland

## Abstract

The framework provided by the Millennium Development Goals includes maternal health as an area of priority. Postnatal depression (PND) is a serious public health issue because it occurs at a crucial time in a mothers' life, can persist for long periods, and can have adverse effects on partners and the emotional, behavioural, and cognitive development of infants and children. Internationally, public health nurses (PHNs) are key professionals in the delivery of health care to mothers in the postpartum period, and international research collaborations are encouraged. Two researchers from the European Academy of Nursing Science (EANS) identified a need to collaborate and strengthen research capacity and discussion on postnatal depression, a public health nursing issue in both countries. Within the context of public health and public health nursing in Ireland and Norway, the aim of this paper is to present a discussion on the concept of PND, prevalence, and outcomes; screening issues for PHNs; and the research evidence of the benefits of social support in facilitating recovery for new mothers.

## 1. Introduction

The WHO-UNFPA [1] has clearly identified maternal mental health as fundamental in attaining the Millennium Development Goals. Postnatal depression (PND) is a significant public health issue, occurring during the perinatal period which is a time of intense change and transition for women. Distinguishing between a natural response to motherhood and symptoms of PND can be difficult both for new mothers and their families [2, 3]. Detection of and intervention in postnatal depression is crucial to the well-being of mothers, their infants, partners, and families. It occurs at a critical time in a mothers' life and can persist for long periods. It can have adverse effects on partners and on emotional and cognitive development of infants and children [4–6]. Public health nurses (PHNs) all over the world have a major role in supporting families with new born babies, and a key concern for public health nursing is the framework provided by the Millennium Development Goals which includes improving maternal health [1]. Many cases of postnatal depression are not detected [7] as there is no international agreement on screening for postnatal depression. There are opinions that the screening instruments do not meet the WHO criteria for when screening should be performed [8]. The Marcé Society for Perinatal Mental Health is an international society for the understanding, prevention, and treatment of mental illness related to childbearing [9]. There is a growing view within the society in favour of undertaking universal psychosocial assessment in perinatal women, as long as it takes place within an integrated care model [10]. Ireland and Norway have many similarities from a geographic, demographic, and public health care model and public health nursing perspectives. The PHN is the primary health care professional providing care to women in the postnatal period in both Ireland and Norway.

The European Academy of Nursing Science (EANS) is a forum for connecting nurse/midwife scientists within Europe through scholarship and research [11]. It offers opportunities to test innovative ideas, pool expertise, and strengthen research capacity in line with the objectives of the European Research Area. Researchers may collaborate across participating countries on any subject which demonstrates a need for international cooperation. To draw attention to important common challenges for nurses, this collaborative research has great significance. The authors met at an EANS conference in the summer of 2012 and identified a need to collaborate and strengthen research capacity and discussion on postnatal depression as a public health nursing issue in both countries. The aim of this paper is to present a discussion on the concept of PND, prevalence, and outcomes; similarities and differences in public health and public health nursing models in Ireland and Norway; research evidence on identification and screening issues for PHNs; and the benefits of social support in facilitating recovery for new mothers. Whilst it is acknowledged that other health care professionals such as midwifes, social workers, and psychologists also contribute to care of women with PND, the focus of this paper is on the role of the PHN. This paper will contribute to the discourse on PND and PHNs contribution in identification and treatment in the context of primary health care internationally. 

## 2. Prevalence and Outcomes Related to Postnatal Depression

PND in women usually occurs 4–6 weeks after birth, and international studies find that between 8% and 15% of mothers are affected by this condition [12–15]. However, in some studies, the prevalence of postnatal depression ranges from zero to almost 60% [16], and the prevalence rates vary across and within countries, from as low as 4.4% at 12 months to as high as 73.7% [17]. In some countries, there are few reports of PND, whereas in other countries reported postnatal depressive symptoms are very prevalent. Prevalence rates reported from Ireland have also varied from 11.4% to 28.6% [18] with the most recent study with first-time mothers reporting prevalence rates of 13% at 6 weeks and 10% at 12 weeks [13]. Four Norwegian studies show prevalence between 8.9% and 16.5% [19–22]. These figures indicate a serious clinical issue for PHNs providing postnatal care to new mothers in the community.

There may be many reasons for this variation in prevalence which include using different screening assessments, using varying cut-off scores (10–13) on the Edinburgh Postnatal Depression Scale (EPDS) [23], assorted timescales (6–12 weeks postpartum), and different samples. For example, one study included a high representation of a sample of mothers with previous history of depression [24]. However, it is well documented that postnatal depression affects at least 10% to 15% of all mothers within the first postpartum year [2, 3, 16, 25]. Thus, several thousand women are affected by this condition each year and this should be an important issue for public health services. This condition has well-documented health consequences for the mother, child, and family [3]. 

Women who have PND are significantly more likely to experience future episodes of depression, and infants and children are particularly vulnerable because of impaired maternal-infant interactions and significant cognitive and emotional development [3, 5]. The nature and symptoms of PND are characterised by tearfulness, fatigue, anxiety, despondency, and excessive anxiety over the baby [23]. An indication of PND is a low mood that causes every day to be experienced as heavy and grey. Some women experience loss of control over their existence, which can lead to an increasing feeling of unease, irritability and outbreaks of anger, inability to cope, and thoughts of suicide. Depression ranges from mild, temporary episodes of sadness to severe, persistent depression [2]. Depressed mothers report higher parenting stress than nondepressed mothers [26, 27], and maternal depressive symptoms might also contribute to unfavourable parenting practices [28] which can adversely affect child growth and development and thus a concern for PHNs. 

## 3. Public Health Care and Public Health Nursing Services

Ireland and Norway have many similarities from a geographic and demographic perspective and both have a strong commitment to primary care and public health. Both countries have similar sized populations, but economically there are differences in relation to poverty, life expectancy is lower, and inequalities are higher in Ireland [29]. The public health system in Ireland is a two-tier system where public and private sectors exist and is governed by the Health Act of 2004 [30]. Following this legislation, the Health Service Executive was established and is responsible for providing health and personal social services to the population. The public health system has a number of on-going issues which could have an impact on primary care services. These include long waiting lists; over capacity on hospital beds; patients awaiting admission on trolleys in the emergency departments; moratorium on staff recruitment leading to staff shortages. Ireland's two-tier health care system has failed in many respects to deliver adequate, fair, and equitable services to meet people's needs [31]. Not all citizens in Ireland have free health care at the point of delivery as it is based on income. Many health care payment schemes operate such as the General Medical Services (GMS) card, Pay Related Social Insurance (PRSI), and drug payment scheme. Nearly 40% of the population are covered by a medical card or a GP visit card [32]. Mental health services have not been prioritised by government and the quality of services lag behind international best practice. There is an ongoing recognition for the need for a shift from the medical model and in-patient treatment to a holistic model of care with recovery and community services at its core [33, 34]. 

In contrast to Ireland, Norway has universal health care for its entire population and free health care at the point of delivery. Municipalities are responsible for managing the services within Norwegian laws and regulations [35]. The Norwegian government has recognized the need for public health services to address mental health issues for women during pregnancy and after childbirth and acknowledges that well-child clinics are an especially suited arena for preventive mental and social work [36]. In both “The women's health strategy” in St. meld. nr. 16 (2002-2003) [37] and the government's “Strategic plan for the mental health of children and adolescents…” is the commitment to expand and strengthen support for women in this period of their lives. There is also a wish to increase research on women's mental health during pregnancy and birth [38], which also reflects the ethos of the Vision for Change strategy document in Ireland [33]. In a recent report from Australia [39], perinatal depression is estimated to cost the Australian economy $433.52 million in 2012, in financial costs only ($4,509 per person with perinatal depression). In addition to the financial costs, perinatal depression equates to a loss of 20,732 disability-adjusted life year DALYs in 2012, which represents a significant disease burden. 

There are no comparable figures available for Ireland and Norway, but it is reasonable to assume similar costs to their economies. Guidelines for treatment of postpartum mental disorders are lacking in both Ireland and Norway [33, 40, 41], and resources have not been increased either in Norway [36, 42] or in Ireland [29]. Furthermore, hospital stay for women after delivery has been dramatically shortened in the last decades, from previous 5–7 days to currently 1-2 days. Since primary health care has not received the required amount of resources [33, 43], support for new families is significantly impaired. There is need for clinical nursing service improvement both from a resource and evidence based perspectives specifically for the identification and management of PND. 

In Ireland and Norway, public health nurses (PHNs) are geographically based and provide a nursing service to new mothers and their infants in the community. Ireland has generalist public health nurses, which means they care for all persons within their defined geographic area from the cradle to the grave [44]. In contrast, PHNs in Norway are specialists and are responsible for preventive services provided to infants, children, adolescents, and their families [45]. Maternity services are free which entitles every woman to General Practice (GP) and hospital obstetric services. In general, midwives are employed to work in the hospital system with some regions having minimal community based service for up to 10 days postpartum. The work of PHNs consists of health promotion and primary prevention, which means promoting mental and physical health as well as good social and environmental conditions and preventing disease, injury, and disability [44, 46]. PHNs in Ireland are mandated to visit all new mothers within 48 hours of discharge from hospital, and similar to PHNs in Ireland are mandated to visit all new mothers within 48 hours of discharge from hospital, and similar to PHNs in Norway who offer home visits within the early weeks after birth and attendance at well baby clinics until the child is four years [40] or school going age [44]. Given the short length of stay at the maternity wards, this home visit is especially important to support the new family. Support and information from the PHN at the home visit can have a preventive effect on depressive symptoms in postpartum women [20, 47].

## 4. Identification of Postnatal Depression 

On a very basic level, Norway has far more PHNs devoted specifically to public health issues, with one client group, compared with PHNs in Ireland providing services to all client groups with a preventative and curative remit. In Norway, there are 2069 PHNs employed in municipal family health clinics and school health services, and in Ireland there were 1702 PHNs employed in the Irish Health Service Executive [29]. PHNs in both countries have the most contact with mothers in the postpartum period and therefore are in a prime position to assess for postnatal depression and facilitate and help mothers to mobilise support from their social network and also to provide support when none are available. In Norway, recent reports suggest that there is not enough research of satisfactory quality available to give recommendations for how to work with PND in the municipalities [8, 48, 49]. In February 2013, The National Council for Priority Setting in Health Care in Norway [8] recommended that screening for postnatal depression should not be introduced on a national basis at the present time. The decision was based on that the EPDS screening does not meet the WHO criteria for when screening should be performed. However, the recent position paper by the Marcé Society recommends undertaking universal psychosocial assessment in perinatal women, as long as it takes place within an integrated care model [10]. In Ireland, recommendations are made for interventions to address PND which may have a wide range of socioeconomic benefits, extending well beyond the impact of the intervention on the mother [33]. Screening for PND is currently not a routine component of the PHN postnatal visit, and thus, many women may not be assessed [50].

There is growing evidence that PND can be effectively treated and possibly prevented [27, 51–53]. However, according to Dennis [54] it is still undetected or untreated in many women. Although a number of tools (essentially self-report questionnaires) have been developed for the detection of depression, only eight studies assess their use in the postnatal period [55]. Only one of these, the Edinburgh Post Depression Scale (EPDS) [23], has been used in a sufficient number of studies to make a judgement on its usefulness. Recent studies [14, 25, 27, 28, 51, 53] indicate that EPDS can be a useful tool to detect PND in women. Cox et al. [23] developed this self-rating scale for detecting depressive symptoms among women who have just given birth. The scale has been translated into several languages. The scale considers the intensity of depressive symptoms that are present in the previous seven days. EPDS has been used both in clinical settings and in epidemiological studies and is generally well accepted by women [56, 57]. Although the sensitivity and specificity vary across languages and cultures, the sensitivity and specificity of the EPDS have been satisfactory in several studies [2, 15, 21, 58]. The form is described as a reliable screening tool [12] and has been recommended for screening of postnatal women [15, 59]. There has been much debate in the literature as to the suitability of using the EPDS in clinical practice for screening for PND. This reluctance is primarily related to the EPDS having reasonable sensitivity but lower specificity, and thus, positive predictive value is poor. This means that many women who do not have PND are being told of the possibility that they have the condition and then could be subject to further investigation, placing an increased and wasteful burden on resources. However, it is important to be aware that the EPDS is a screening instrument that indicates the possible presence of depression and not a diagnostic tool. To determine a clinical diagnosis of PND, it is necessary to use the EPDS, followed by a clinical assessment and an interview [2]. Thus, the clinical assessment done by the PHN after the EPDS is decisive of further followup. PHNs have described EPDS as a door opener for talking to new mothers about their mental health [52]. According to Seeley [60], the EPDS is only as good as the person using it. Similarly, using the Whooley et al. [61] questions plus the additional Arroll et al. [62] question has also demonstrated poor positive predictive value. Nonetheless, the Current NICE [63] guidelines recommend using them. Although little specific evidence exists for their use in the perinatal period, their ease of use and reasonable sensitivity and specificity, particularly if combined with the additional help question from Arroll et al. [62], suggest that their use in routine care may be practical and acceptable. The questions are simple screening methods which can detect postnatal depression and lead to a subsequent referral for a full clinical assessment followup. This screening technique is an opportunity to screen without the need for a more formal assessment. However, all postnatal depression screening and assessment must be combined with a treatment chain and systematic referral procedures [2, 10, 64]. Public health nurses have the most contact with mothers and new babies in the postpartum period and therefore are in a prime position to assess for PND and provide support. According to Negron et al. [65], it is important to identify social support resources needs of new mothers to facilitate their transition to motherhood and recovery after childbirth. 

## 5. Social Support

International and national policy documents suggest that social support is necessary for maternal and infant well-being and facilitates women's transition to motherhood. In previous research, mothers in the postnatal period have reported help received from their partners and mothers, both with household chores and infant care, to be of great importance to them. Providing support for mothers in caring for their infants in the postnatal period is an important concern for nurses in the community, because research has shown that social support can facilitate women's transition to motherhood [66], some of whom find the transition psychologically stressful. Furthermore, previous research has indicated that social support from partners, maternal mothers and peers [67], and home visits from nurses [22, 68] have reduced postnatal depressive symptoms. Within the Irish context, given the importance of social support in facilitating transition to motherhood, Leahy-Warren [69] conducted research with first-time mothers (*n* = 99) exploring the relationship between social support and confidence in infant care practices at 6 weeks postpartum. Findings revealed that support in the guise of mothers' receiving positive affirmation with caring for their infant had a significant influence on their confidence in caring for their infants. Mothers' revealed that the sources of this type of support were their partners and own mothers. Results also showed that public health nurses and maternal mothers were the primary source of informational support. Therefore, it is essential that nurses facilitate the identification of individual mothers' sources of support and continue to provide them with information that is relevant and appropriate. 

A more recent Irish study examined the relationship between postnatal depression, maternal parental self-efficacy (confidence), and postnatal depression during the first 3 months postpartum with a large sample of first-time mothers (*n* = 512) [14, 50]. The results showed that at 6 weeks, significant relationships were found between functional social support and postnatal depression and informal social support and postnatal depression. This means that support received from mothers' partner, own mother, family, and friends positively influenced postnatal depressive symptoms at 6 weeks. The types of support that were significant were informational, instrumental (hands-on help), emotional (caring) and appraisal (positive affirmation). Findings also revealed that the higher the level of maternal parental self-efficacy (confidence) the lower the level of depressive symptoms. This means that mothers who have confidence in their own ability to care for their infants are less likely to have postnatal depressive symptoms. Nurses need to be aware of and acknowledge the significant contribution of social support, particularly from family and friends in positively influencing first-time mothers' mental health and well-being. 

The best predictors of postnatal depression at 12 weeks were at-birth professional support and emotional support. What this means is that mothers who received low levels of professional support at birth were 3.24 times more at risk of PND at 12 weeks than mothers who received high levels of professional support. Furthermore, there was an elevated risk (2.92 times) of PND at 12 weeks in mothers with low emotional support, compared with those who received high emotional support at birth [18, 44]. In a study with first-time mothers (*n* = 271), when their babies were 3 months old, Tarkka et al. [70] showed that social support and support from public health nurses were important factors in first-time mothers coping with child care. Similar findings were reported from Taiwan, where findings revealed that nursing interventions enhanced women's (*n* = 122) social support and decreased their PND [71]. Razurel et al. [72] interviewed 60 women six weeks after the birth of their first child. The new mothers expressed the need to be supported and counselled when problems arose and regretted the lack of long-term postpartum support. Gao et al. [73] compared the prevalence of depression in the postpartum period and its relationship with perceived stress and social support in first-time mothers and fathers. In this cross-sectional study with a sample of 130 pairs of parents, they found that perceived stress, social support, and partner's depression were significantly associated with depression in new mothers and suggest that counselling, support, and routine screening for depression should be provided to both mothers and fathers. A qualitative study using focus groups of women (*n* = 33) participating in a postpartum depression randomised controlled trial explored their experiences of social support in the postpartum period [65]. One of the main themes identified were mothers' major needs and social support expectations including providers of social support. Mothers indicated that support from partners and family was expected and should be provided without asking. Furthermore, findings indicated that identifying support needs and expectations of new mothers is critical for mothers' recovery after childbirth. These findings signify the need for public health nurses to be mindful of the importance of support for mothers in the early postnatal period. Recovery can be facilitated by helping mothers identify the types of supports they need and who is best from their social network to provide specific supports. PHNs can contribute by facilitating and helping mothers to mobilise support from within their social network. 

Glavin [47] discusses a model for prevention, identification, and treatment for PND in a Norwegian municipality. The PHNs in the intervention municipality undertook specific training related to PND [27, 52]. In this study, 2227 women participated, 437 in the control group and 1790 in the intervention group. At the home visit two weeks postpartum, the PHNs in the intervention group gave information (both written and oral) about PND and encouraged the mother to contact the well-child clinic before the first appointment if she felt depressed. A significant difference in PND symptoms was detected between the two groups at six weeks postpartum. This indicates that information and support can prevent some cases of PND with better outcomes for maternal and infant health and well-being [22]. The PHNs in the intervention municipality used the EPDS followed by a clinical assessment and an interview to assess all mothers for PND at six weeks postpartum. The assessment was followed up by supportive counselling sessions with a PHN for women in need of that. A total of 228 women, 64 in the control group and 164 in the intervention group, had an EPDS score ≥10 at six weeks. The women who received supportive counselling sessions showed a significant decrease in depression score compared to the usual care group up to 12 months postpartum [74]. The study by Glavin et al. [27, 47, 74] showed an effect on depressive symptoms among depressed women as well as among the nondepressed up to 12 months after delivery, and the results are supported by other studies [51, 53]. In a prospective cluster trial, randomized by GP practice with 1474 interventions and 767 control women, Morrell et al. [53] and Brugha et al. [51] used EPDS and trained health visitors to assess for PND and give supporting counselling sessions to mothers in need of that. They also reported a decrease in the PND scores among women who received support from health visitors. A review including fifteen trials, involving over 7600 women, Dennis and Creedy [75] reported that home visits after birth by public health nurses or midwives helped to prevent PND. Thus, several studies indicate that support from the PHN may have a preventive effect on PND in women.

## 6. Conclusion

The prevalence of PND at 10–15% is a serious public health issue and consequently a public health nursing clinical concern in the community. The adverse consequences of PND for mothers and their families necessitate the need for PHNs to identify those at risk. Public health care in the guise of primary care in Ireland and Norway is an ideal integrated model of care in the community in which universal screening could be achieved by PHNs with appropriate and adequate resources. Research evidence has demonstrated the significant beneficial effects of PHN support visits and facilitation and mobilisation of social supports from mothers' social network. Priority needs to be given at a strategic level in both countries to resource a perinatal mental health strategy, embedded in public health policy to ensure that universal psychosocial assessment in perinatal women is undertaken within an integrated care model. The prevention, detection, and treatment of this condition in women are crucial. This needs to be considered given the benefits to the individual, the family, the community, the health care profession, and financial costs to each country.
